# Asymmetric Schottky Barrier-Generated MoS_2_/WTe_2_ FET Biosensor Based on a Rectified Signal

**DOI:** 10.3390/nano14020226

**Published:** 2024-01-20

**Authors:** Xinhao Zhang, Shuo Chen, Heqi Ma, Tianyu Sun, Xiangyong Cui, Panpan Huo, Baoyuan Man, Cheng Yang

**Affiliations:** 1 School of Physics and Electronics, Shandong Normal University, Jinan 250014, China; zhangxh0050@163.com (X.Z.); shuochen1119@gmail.com (S.C.); heqima9@163.com (H.M.); s15169093367@126.com (T.S.); CXY2023025549@outlook.com (X.C.); hpp6780@163.com (P.H.); 2 Shandong Provincial Engineering and Technical Center of Light Manipulations, Shandong Normal University, Jinan 250014, China

**Keywords:** Schottky junction, field-effect transistor, biosensor, rectified signal, ultra-sensitive detection

## Abstract

Field-effect transistor (FET) biosensors can be used to measure the charge information carried by biomolecules. However, insurmountable hysteresis in the long-term and large-range transfer characteristic curve exists and affects the measurements. Noise signal, caused by the interference coefficient of external factors, may destroy the quantitative analysis of trace targets in complex biological systems. In this report, a “rectified signal” in the output characteristic curve, instead of the “absolute value signal” in the transfer characteristic curve, is obtained and analyzed to solve these problems. The proposed asymmetric Schottky barrier-generated MoS_2_/WTe_2_ FET biosensor achieved a 10^5^ rectified signal, sufficient reliability and stability (maintained for 60 days), ultra-sensitive detection (10 aM) of the Down syndrome-related DYRK1A gene, and excellent specificity in base recognition. This biosensor with a response range of 10 aM–100 pM has significant application potential in the screening and rapid diagnosis of Down syndrome.

## 1. Introduction

Low-dimensional materials-based field-effect transistor (FET) biosensors have the advantages of high sensitivity, fast detection, miniaturization, low cost, and compatibility with integrated circuits [1,2,3]. However, the commonly used signals in FET biosensors are referred to as “absolute value signals”. These include Dirac voltage, threshold voltage *V*_th_, source-drain current I_ds_, and others [4,5,6,7]. These “absolute value signals” directly collect and process the specific changes in biomolecules. Although these signals carry the biomolecules’ charge information [8,9,10], they have the drawbacks of incomplete, unstable, and unreliable detection. Kulkarni et al. reported that the detection range of FET-based biosensors was limited by the Debye length due to the Debye screening effect. Consequently, molecular charge information outside the Debye length cannot be detected [11]. Sadighbayan et al. reported that ions in water or an electrolyte solution affected the surface potential or electric field of the sensor, causing fluctuations in the “absolute value signal”. The fluctuations made the stable detection of the charge information of biomolecules challenging [12]. Xu et al. reported that non-target molecules on the sensor surface can generate non-specific signals, affecting the charge density or potential on the sensor surface. This non-specific signal makes the detected “absolute value signal” unreliable [13,14].

A series of signal optimization models and signal processing methods for the “absolute value signals” have been proposed by researchers to accurately express the charge information carried by biomolecules. Fathi-Hafshejani, Parvin and coworkers combined density functional theory (DFT) and the collected “absolute value signals”. They optimized the calculation model of the interaction energy and charge transfer between MUA (mercaptoundecanoic acid) and WSe_2_, achieving the rapid and sensitive detection of the novel coronavirus (SARS-CoV-2) [15]. By adding a MoS_2_ layer on graphene, Chen et al. suppressed the interference caused by the Debye screening effect on charge information detection. Consequently, they achieved the high integrity of charge information detection under physiological environment conditions [16]. Hajian, Reza et al. used the double exponential function and linear regression model to determine the relationship between the signal change curve and DNA concentration, and then cross-checked the theoretical results with the experimental “absolute value signals” to improve the detection stability [17]. Sarah Balderston et al. identified an insurmountable hysteresis phenomenon in measuring the transfer characteristic curve. They proposed a signal-processing model based on the average transconductance of small-range scanning gate voltage to minimize the interference of the hysteresis phenomenon on the measurement results, achieving single-base mutation detection in the BRCA1 and KRAS genes [18]. These models and strategies have significantly promoted signal accuracy. However, due to the inherent defects of the sensing materials, measurements still suffer from the interference of the hysteresis phenomenon while determining the influence of biomolecules on the intrinsic electric field of the sensing materials.

This study, for the first time, reports a strategy of using a “rectified signal” in the output characteristic curve instead of the “absolute value signal” in the transfer characteristic curve. A Schottky barrier-based MoS_2_/WTe_2_ FET biosensor was prepared to generate a “rectified signal” in the output characteristic curve. The low work function semimetal WTe_2_ electrode replaced the Au electrode and formed a Schottky junction with MoS_2_, reducing the Schottky barrier height at the WTe_2_/MoS_2_ interface and increasing carrier mobility. The Schottky barrier difference between the WTe_2_/MoS_2_ electrode and MoS_2_/Au electrode caused the rectification ratio. Furthermore, the MoS_2_/WTe_2_ FET biosensor was used as a sensing platform to detect the Down syndrome-related DYRK1A gene, achieving a detection limit of 10 aM and high specificity. The linear response range was 10 aM–100 pM, indicating that the MoS_2_/WTe_2_ FET biosensor has broad application potential in the screening and rapid diagnosis of Down syndrome.

## 2. Materials and Methods

### 2.1. Materials

PBS (pH~7.0–7.2), 1-pyrenebutanoic acid succinimidyl ester (PBASE), DMSO, and ethanolamine were procured from Aladdin Co., Ltd. (Shanghai, China). SiO_2_/Si substrates, bulk MoS_2_, 1T’-WTe_2_, blue film tape, and PDMS (polydimethylsiloxane) film were purchased from Shanghai Onway Technology Co., Ltd. (Shanghai, China). Other reagents were procured from Sinopharm Chemical Reagent Co., Ltd. (Shanghai, China). Deionized water (DI water) was collected from a Millipore water purification system (Milli-Q Direct8). The partial DNA/RNA sequences were purchased from Sangon Biotech (Shanghai) Co., Ltd. (Shanghai, China) (Table S1).

### 2.2. Device Fabrication

MoS_2_ and 1T’-WTe_2_ films were peeled off from their crystal blocks using blue film tape and then transferred to the PDMS film using a two-dimensional material transfer platform. Finally, MoS_2_ and 1T’-WTe_2_ films with the optimal thickness, size, and morphology were transferred to the SiO_2_/Si substrates with pre-lithographed electrodes, forming 1T’-WTe_2_/MoS_2_ devices (Figure S1).

### 2.3. Device Functionalization and Immobilization

Before the detection of the target DNA molecule, the constructed MoS_2_/WTe_2_ needed to be functionalized and immobilized. PBASE was selected to functionalize MoS_2_ and immobilize the probe DNA as a linker between MoS_2_ and probe DNA. PBASE, when dissolved in DMSO, stacks its pyrene group on the surface of MoS_2_, binds to MoS_2_, and immobilizes the probe DNA via the coupling reaction between the amine of the probe DNA and the amine-reactive succinimide group of PBASE (Figure S2). During functionalization, the PBASE solution, placed in an Eppendorf tube (EP tube), reacted with the MoS_2_/WTe_2_ FET biosensor at 37 °C for 20 min in a constant temperature box to deposit the pyrene groups in PBASE on the MoS_2_ surface by π-π stacking. After reaction completion, the unreacted samples on the surface were washed off with DMSO solution. During the probe DNA immobilization, the probe DNA solution (1 pM) was placed in an EP tube and reacted with the MoS_2_/WTe_2_ FET biosensor at 37 °C for 2 h in a constant temperature box to immobilize the probe DNA via a conjugation reaction between the amine group of the probe DNA and the amine-reactive succinimide group of PBASE. The shift of the Mo3d peak and S2p peak positions confirmed that PBASE and the probe DNA were successfully combined on the MoS_2_/WTe_2_ surface (Figure S3). After reaction completion, the unreacted gene samples on the surface were washed off with 1×PBS solution. In the target DNA detection process, 200 μL of the DYRK1A gene sample was placed in an EP tube, followed by a reaction with the MoS_2_/WTe_2_ FET biosensor at 37 °C for 2 h in a constant temperature box to allow more target DNA to diffuse to the MoS_2_ surface and be captured by the probe DNA on the surface. After completion, the unreacted gene samples on the surface were washed off with 1×PBS solution, and the performance of the MoS_2_/WTe_2_ FET biosensor was tested after drying.

### 2.4. Characterization

The electrical properties of the MoS_2_/WTe_2_ FET biosensor were determined using a Keithley 4200-SCS semiconductor parameter analyzer at room temperature and in atmospheric pressure, dry, dark, and well-ventilated conditions. A constant stride interval of 20 mV was applied to the V_DS_–I_DS_ curve. The structure and morphologies of the prepared samples were analyzed using SEM (Zeiss Gemini Ultra-55, 3.0 kV, Oberkochen, Germany) and EDS. AFM was used to determine the roughness of the MoS_2_/WTe_2_ surface. The Raman spectrometer used in this study was a Horiba HR Evolution 800 with a 532 nm excitation laser. The XPS characterization was conducted using a Thermo Fisher Scientific Escalab 250Xi instrument (Waltham, MA, USA) with an Al K X-ray source at 150 W and a spot size of 500. The spectra were acquired with an operating voltage of 12.5 kV and a spectrometer pressure of 8 × 10^−10^ mbar. The XPS spectra were calibrated by the peak of C 1 s at 282 eV, normalized by the baseline level, and curve-fitted by smart function.

## 3. Results and Discussion

### 3.1. The Advantages and Generation Mechanisms of the Rectified Signal

The rectified signal is a kind of response signal, which is composed of two currents with opposite directions. It effectively reflects the specific changes in the biomolecules. Taking the current variation (ΔI) and rectification ratio variation (ΔR) as examples, the accuracy of the “absolute value signal” and “rectified signal” can be discussed in detail.

**Figure 1:**
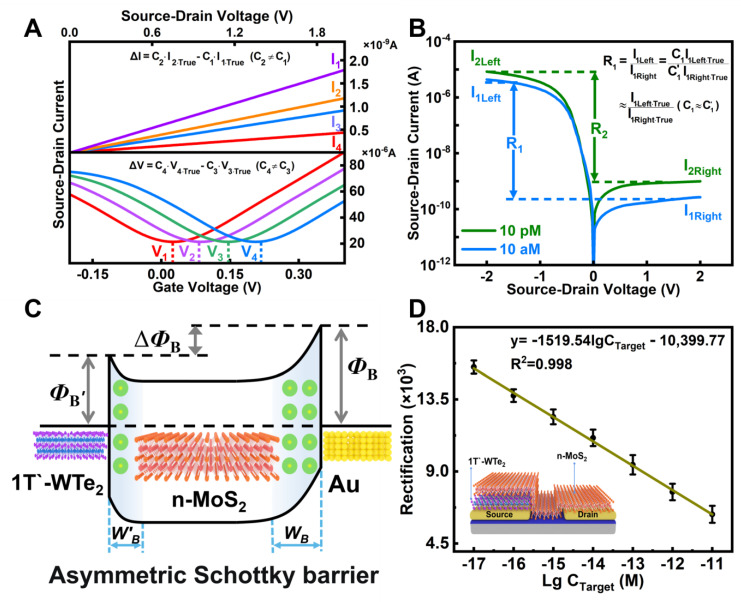
(**A**) Current–voltage images using the source-drain current, Dirac voltage, and other absolute value signals as response signals. (**B**) Current–voltage image when the rectification ratio, a rectified signal, is used as a response signal. (**C**) The band diagram of the structure generating the rectification ratio signal. (**D**) The linear relationship between the rectification ratio signal and the concentration of biomolecules. The inset shows a schematic diagram of the structure.

Figure 1A shows that, when the “absolute value signal” (such as source-drain current) is used as the response signal, which can be expressed as [19]

(1) $$\Delta I=I_{2}-I_{1},$$

$I_{1}$ and $I_{2}$ are the measured source-drain currents at different biomolecular concentrations.

(2) $$I_{1}=C_{1}\cdot I_{1\cdot \mathrm{True}};\;I_{2}=C_{2}\cdot I_{2\cdot \mathrm{True}},$$

$I_{1\cdot \mathrm{True}}$ and $I_{2\cdot \mathrm{True}}$ are the accurate values of the source-drain current at different biomolecular concentrations without interference from external factors. The constant C_i_ (i = 1, 2) represents the interference coefficient of external factors at different biomolecular concentrations. Since $I_{2}$ and $I_{1}$ are “absolute value signals” at different biomolecular concentrations, each external factor affects them differently ($C_{2}\neq C_{1})$, which implies that the charge information carried by biomolecules cannot be accurately determined by using an “absolute value signal” as a response signal.

Figure 1B shows that, when the “rectified signal” is used as a response signal, the rectification ratio can be expressed as [20]:

(3) $$\Delta R=R_{2}-R_{1},$$

where R_1_ and R_2_ are the rectification ratios at different biomolecular concentrations.

(4) $${\;R}_{1}=\frac{I_{1\cdot \;\mathrm{Left}}}{I_{1\cdot \;\mathrm{Right}}}=\frac{C_{1}\cdot I_{1\cdot \;\mathrm{Left}\cdot \mathrm{True}}}{C_{1}^{’}\cdot I_{1\cdot \;\mathrm{Right}\cdot \mathrm{True}}}\;\approx \;\frac{I_{1\cdot \;\mathrm{Left}\cdot \mathrm{True}}}{I_{1\cdot \;\mathrm{Right}\cdot \mathrm{True}}}(C_{1}\;\approx \;C_{1}^{’}),$$

(5) $$R_{2}=\frac{I_{2\cdot \;\mathrm{Left}}}{I_{2\cdot \;\mathrm{Right}}}=\frac{C_{2}\cdot I_{2\cdot \;\mathrm{Left}\cdot \mathrm{True}}}{C_{2}^{’}\cdot I_{2\cdot \;\mathrm{Right}\cdot \mathrm{True}}}\;\approx \;\frac{I_{2\cdot \;\mathrm{Left}\cdot \mathrm{True}}}{I_{2\cdot \;\mathrm{Right}\cdot \mathrm{True}}}(C_{2}\;\approx \;C_{2}^{’}),$$

I_i Left_ (i = 1,2) and I_i Right_ are the source-drain currents under the sweep negative and positive voltages, respectively. Since $I_{i\cdot \;\mathrm{Left}}$ and $I_{i\cdot \;\mathrm{Right}}$ are measured at the same biomolecular concentration, they should be affected by external factors approximately equally ($C_{i}\approx C_{i}^{’}$), which means that $R_{2}$ and $R_{1}$ values are approximately accurate. Thus, the variation in the “rectified signal” ($\Delta R=R_{2}-R_{1})$ is approximately accurate. Using the “rectified signal” as a response signal accurately reflects the charge information carried by biomolecules.

A MoS_2_/WTe_2_ FET biosensor with an asymmetric Schottky barrier was constructed to measure the rectification ratio signal. Figure 1C shows the schematic diagram of its energy band structure. High-quality n-type MoS_2_ and low-work function semimetal 1T′-WTe_2_ were obtained by mechanical exfoliation and built into heterojunctions by a transfer platform [21]. In the MoS_2_/WTe_2_ FET biosensor, MoS_2_ forms asymmetric Schottky junctions with 1T′-WTe_2_ and Au, with barrier heights of $\Phi_{B}^{’}$ and $\Phi_{B}$ and barrier widths of $W_{B}^{’}$ and $W_{B}$, respectively. The Schottky barrier height can be obtained from the Schottky–Mott relation [22]:

(6) $$\Phi_{B}=\Phi_{M}-\chi_{S},$$

where $\Phi_{B}$ is the Schottky barrier height, $\Phi_{M}$ is the work function of the metal electrodes, and $\chi_{S}$ represents the electron affinity of the n-type semiconductor. The Schottky barrier width can be obtained by the following formula [23]:

(7) $$W=\sqrt{\frac{2{ℇ}_{s}V_{\mathrm{bi}}}{qN_{A}}},$$

where ${ℇ}_{s}$ is the dielectric constant of the semiconductor; $V_{\mathrm{bi}}=\left(\Phi_{M}-\Phi_{S}\right)/q$ is the built-in potential of the Schottky junction; q is the elementary charge; and $N_{A}$ is the bulk charge density of the channel material. 

Because of the lower work function of WTe_2_ compared to that of Au, the Schottky barrier height and width of the MoS_2_/Au junction is larger than those of the MoS_2_/1T′-WTe_2_ junction in the WTe_2_/MoS_2_/Au FET biosensor. The asymmetric Schottky barrier height between the two junctions leads to the generation of a rectification ratio signal in the heterojunction FET. As we know, the smaller the barrier width, the larger the tunneling current. In addition, the widths of the two junctions are different; thus, the smaller the W, the larger the tunneling current. Then, the tunneling currents of the two junctions are definitely not the same, which finally causes source-drain current changes at both ends, resulting in rectification ratio changes, as shown in Figure 1D.

### 3.2. Schematic Diagram of the MoS_2_/WTe_2_ FET Biosensor

**Figure 2:**
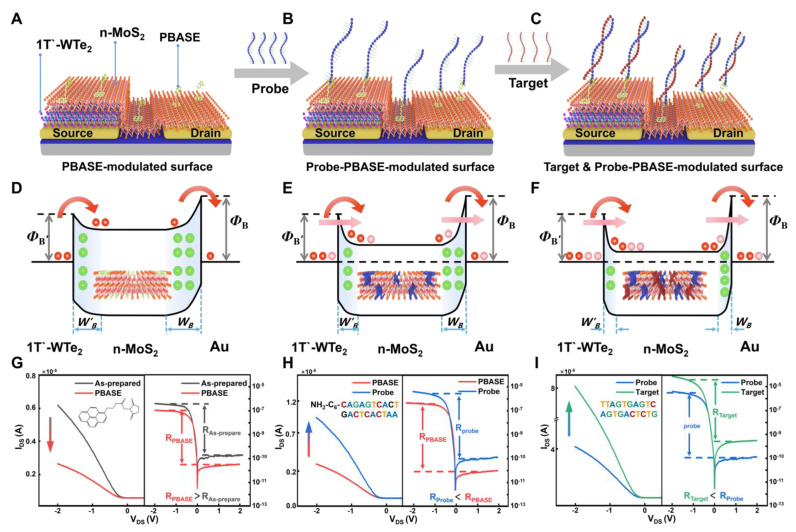
Schematic diagram of the MoS_2_/WTe_2_ FET biosensor. (**A**) Functionalization of the MoS_2_/WTe_2_ surface using the PBASE solution. (**B**) Immobilization of the probe DNA implied an interaction between PBASE and the probe DNA, achieving the binding of the probe DNA and the MoS_2_/WTe_2_ FET biosensor. (**C**) Interaction between the probe DNA and target DNA to form a double-stranded DNA. Schematic diagram of the band gap changes in the MoS_2_/WTe_2_ FET biosensor in the three processes: (**D**) functionalization of the sensing surface, (**E**) immobilization of the probe DNA, and (**F**) binding of the target DNA, where the charge injection mode modulated by PBASE on the surface is thermionic emission (TE), and the charge injection mode modulated by probe-PBASE on the surface and by target & probe–PBASE on the surface is tunneling. Absolute value signal I_DS_ (left) and rectified signal R (right) of the MoS_2_/WTe_2_ FET biosensor in the (**G**) functionalization, (**H**) immobilization of probe DNA, and (**I**) binding of target DNA.

The functionalization of the sensing surface, probe DNA immobilization, and target DNA hybridization were performed using the MoS_2_/WTe_2_ FET biosensor to generate a more stable response signal. In Figure 2A, 1-pyrenebutyric acid succinimidyl ester (PBASE) in the N,N-dimethylformamide solution was used as a linker between MoS_2_ and the probe DNA, ensuring that the probe DNA could effectively bind to MoS_2_ [24]. The pyrene group of PBASE was fixed on the MoS_2_ surface by π-π stacking. The succinimide part of PBASE protruded from the MoS_2_ surface and coupled with the amine group of the 5′-amine-modified DNA probe, as shown in Figure 2B [25]. Next, the target DNA complementary was added to the probe DNA. Consequently, the target DNA was hybridized with the probe DNA by base complementary pairing, as shown in Figure 2C. Notably, the different Schottky barrier widths of the MoS_2_/WTe_2_ FET biosensors can be obtained with the different steps, affecting the probability of tunneling current [26].

In the functionalization process of MoS_2_, the pyrene groups in PBASE fixed on the surface of MoS_2_ by π-π stacking add a small amount of holes to the MoS_2_/WTe_2_ FET biosensor. Hole doping also effectively widens the Schottky barrier width of the MoS_2_/WTe_2_ FET biosensor. At this time, thermionic emission (TE) is the main charge injection mode, as shown in Figure 2D. That is to say, although the decrease in the forward bias current dominated by the MoS_2_/Au junction is small, its relative decrease rate is larger, resulting in the increase in the rectification ratio R, as shown in Figure 2G. In the process of probe DNA immobilization, the succinimide part of the MoS_2_ surface couples with the probe DNA, which fixes the probe DNA on MoS_2_ and adds a small amount of electrons to the MoS_2_/WTe_2_ FET biosensor. Electron doping also effectively reduces the Schottky barrier width of the MoS_2_/WTe_2_ FET biosensor. At this time, the charge injection mode is electron tunneling and TE, as shown in Figure 2E. According to the conclusion, after fixing the probe DNA, the barrier width of the MoS_2_/Au Schottky junction is reduced more and the relative current growth rate is higher than that of the MoS_2_/1T′-WTe_2_ Schottky junction. That is to say, although the increase in the forward bias current dominated by the MoS_2_/Au junction is small, its current growth rate is larger, resulting in the decrease in the rectification rate R, as shown in the right inset of Figure 2H. Similarly, target DNA hybridizes with probe DNA through base complementary pairing, which also adds electron doping to the MoS_2_/WTe_2_ FET biosensor. It increases the current and reduces the Schottky barrier width at the same time, and the barrier width of the MoS_2_/Au junction is reduced more and the relative current growth rate is higher, resulting in the increase in the current and decrease in the rectification ratio R, as shown in Figure 2F,I.

The equation of the Schottky barrier width was used to calculate the change in the barrier width before and after doping. Their effects on the forward, reverse current, and rectification ratio signal were analyzed using Equations (2)–(4) [22]. The rectification ratio signal was found to effectively eliminate the interference of external factors on the molecular signal because it depends only on the ratio of the forward and reverse currents rather than the absolute value of the single-direction current. Therefore, the rectification ratio signal is not affected significantly, and an accurate expression of the charge information carried by biomolecules is obtained.

In addition, the rectification ratio signal not only reflects the changes in both Schottky junctions but also avoids the interference of the hysteresis phenomenon (Figure 2G–I). Since the forward and reverse source-drain currents are affected by the charge information on DNA molecules, the rectification ratio signal can detect weak charge information under the conditions of a low concentration DNA solution. In contrast, the forward or reverse source-drain current, which is used as the response signal, is difficult to separate from the noise signal [27], reducing the sensing sensitivity. Therefore, the rectification ratio signal as a kind of “response signal” is a promising biosensing signal, which can play a key role in biosensors and other sensing devices.

### 3.3. Structure and Characterization Analyses of the MoS_2_/WTe_2_ FET Biosensor

**Figure 3:**
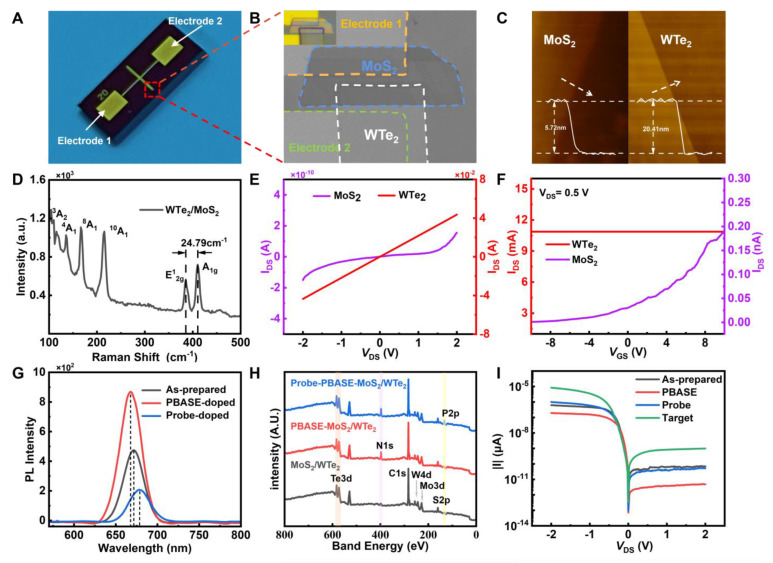
(**A**) A typical MoS_2_/WTe_2_ FET biosensor device. (**B**) SEM image of the MoS_2_/WTe_2_ FET biosensor, with its optical image in the inset. (**C**) AFM images of the MoS_2_ and WTe_2_ films. (**D**) Raman spectra of the MoS_2_/WTe_2_ heterojunction. (**E**,**F**) I_DS_-V_DS_ and I_DS_-V_GS_ characteristics of MoS_2_ and WTe_2_ at room temperature. The inset in (**E**) is the whole I_DS_-V_DS_ characteristics of WTe_2_. (**G**) Photoluminescence spectra with and without biomolecules. (**H**) X-ray photoelectron spectra before adding the target DYRK1A. (**I**) Current output characteristics after adding different biomolecules.

Figure 3A shows an optical image of the fabricated MoS_2_/WTe_2_ FET biosensor, consisting of a MoS_2_/WTe_2_ heterostructure and two electrodes on a SiO_2_/Si substrate; the electrode spacing is 20 μm. Figure 3B shows the SEM image and the optical image of the area marked in Figure 3A. The corresponding EDS mapping of this area, showing the uniform distribution of each element [28], is shown in Figure S4. The EDS mapping shows that the atomic ratio of S to Mo in the MoS_2_/WTe_2_ FET biosensor is 2:1, and the atomic ratio of Te to W is 2:1, indicating the elements’ uniform distribution. Figure 3C shows the image of the MoS_2_/WTe_2_ heterojunction measured by an atomic force microscope (AFM). The line scan profile of the heterojunction height was extracted from the AFM image, while the thicknesses of the MoS_2_ and WTe_2_ flakes were measured to be 5.72 nm and 20.41 nm. The Raman spectrum of the heterojunction (Figure 3D) was measured using a 532 nm laser. The Raman spectrum revealed the characteristic peak E_2g_^1^ representing the in-plane vibration mode was located at 383 cm^−1^ and the characteristic peak A_1g_ representing the out-of-plane vibration mode was located at 407.79 cm^−1^, consistent with the thickness of the MoS_2_ flake [29,30]. The characteristic peaks ^3^A_2_, ^4^A_1_, ^8^A_1_, and ^10^A_1_ of WTe_2_ were located near 116.4, 133.7, 163.9, and 211.8 cm^−1^, respectively, consistent with the crystal structure of WTe_2_ [31].

The electrical properties of MoS_2_ and WTe_2_ were measured [32]. Figure 3E shows the Schottky contact between the MoS_2_ and Au electrodes and the Ohmic contact between the WTe_2_ and Au electrodes. Figure 3F depicts the transfer characteristic curves of the MoS_2_ and WTe_2_ devices at a source-drain voltage V_DS_ = 0.5 V, where MoS_2_ showed conventional n-type semiconductor characteristics [33] and the current of WTe_2_ was unaffected by gate voltage, showing semimetal characteristics [34].

Photoluminescence (PL) and X-ray photoelectron spectroscopy (XPS) were used to verify that this biosensor can be successfully functionalized [35]. After functionalizing with MoS_2_, the PL peak intensity increased, and the peak underwent a blue shift (See Figure 3G). After immobilizing the probe DNA, its PL peak intensity decreased, and the peak red-shifted. The peak intensity enhancement and the blue shift can be attributed to the following: The functionalized MoS_2_ was affected by PBASE, where the electrons, trions A^−^, and foreign holes combined to form several neutral excitons A^0^, increasing the proportion of neutral excitons A^0^ and an enhancement in radiative transition. Consequently, the dominant exciton in the A peak changed from trion A^−^ to neutral exciton A^0^, leading to a PL peak intensity enhancement and the blue shift of the peak position. The peak intensity reduction and red shift can be attributed to the following: The immobilized MoS_2_ was affected by the probe DNA, where foreign electrons and neutral excitons A^0^ combined to form trions A^−^. This was followed by the dominant exciton in the A peak changing from neutral exciton A^0^ to trion A^−^, while the radiative transition weakened, reducing the PL peak intensity and yielding the red shift of the peak position [36]. Figure 3H shows the N1s peak of PBASE and the P2p peak of the probe DNA, confirming the functionalization and immobilization of the probe DNA. The XPS measurements revealed that PBASE and the probe DNA were successively bound to the MoS_2_/WTe_2_ surface. Furthermore, the change in the source-drain current in Figure 3I can be explained by the following reasoning: The adsorption of PBASE on the MoS_2_/WTe_2_ surface caused p-doping, resulting in a decrease in the overall source-drain current. The binding of the probe DNA with PBASE caused n-doping, increasing the source-drain current. Similarly, the complementary pairing of the target DNA with probe DNA caused n-doping, increasing the overall source-drain current [37,38].

### 3.4. Sensing Performance of the MoS_2_/WTe_2_ FET Biosensor

**Figure 4:**
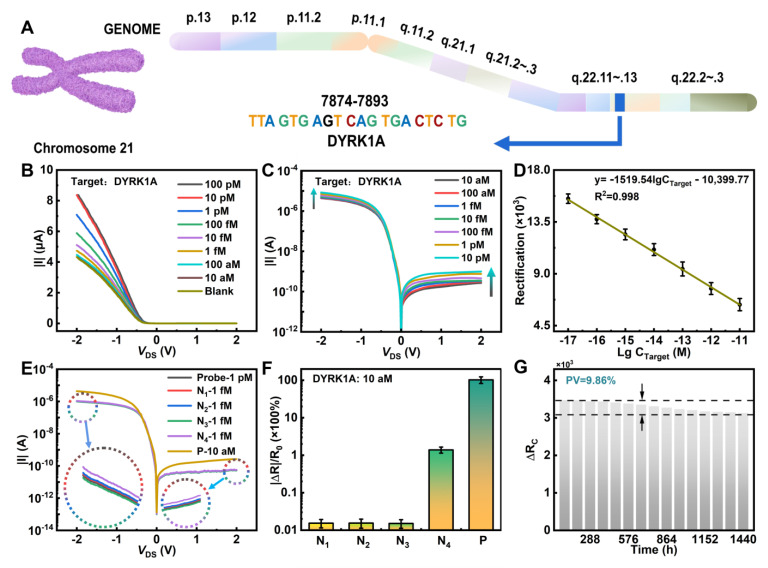
Sensing performance of the MoS_2_/WTe_2_ FET biosensor. (**A**) Target site of Down syndrome in chromosome 21 genome map. (**B**) Response of the output characteristic curve of the target DNA (DYRK1A gene). (**C**) Effect of the target DNA (DYRK1A gene) on the rectification ratio. (**D**) Statistics of the rectification ratio response of the DYRK1A gene. (**E**) Specificity response of the MoS_2_/WTe_2_ FET biosensor. (**F**) A comparison of the target DNA and non-target DNA. (**G**) The rectification ratio signal change values for the two concentrations during the sixty days; the peak to valley value is 9.87%.

Down syndrome [39] is one of the most common chromosomal diseases and one of the main causes of human intellectual disability. The DYRK1A gene on chromosome 21 was chosen as the target DNA to study the sensitivity of the MoS_2_/WTe_2_ FET biosensor (Figure 4A). The functionalized and immobilized MoS_2_/WTe_2_ FET biosensor in different concentrations of the target DNA solution was studied to obtain the relationship between the target concentration and the response signal [40]. Figure 4B shows that the output characteristic curve gradually shifted upward as the concentration of the target DNA (DYRK1A) gradually increased from 10 aM to 100 pM. The current results of the different concentrations of the target DNA (DYRK1A) under positive and negative bias voltages are shown in Figure S5. Since the band gap width of MoS_2_ was not zero [41], the noise signal of the MoS_2_-based FET biosensor was much lower than that of the graphene FET biosensor [42,43]. When the target DNA concentration was as low as 10 aM, the detected source-drain current was much larger than that without adding the target DNA. Therefore, it can be confirmed that the 10 aM target DNA was successfully detected. Figure 4C shows the rectification ratio response with the increasing concentrations of the target DNA, which is attributed to the change in the Schottky barrier width caused by charge doping. Specifically, the change in the source-drain current dominated by the MoS_2_/Au junction is larger than that dominated by the MoS_2_/WTe_2_ junction. Figure 4D shows that the rectification ratio gradually decreased as the concentration increased from 10 aM to 100 pM. Furthermore, the rectification ratio signal had a linear relationship with the concentration gradient ($y=-1519.54\mathrm{lg}C_{\mathrm{Target}}-10,399.77$). These results indicate that the rectification ratio can serve as a kind of detection indicator in the MoS_2_/WTe_2_ FET biosensors.

Four kinds of biomarkers (N_1_–N_4_) diffused into the MoS_2_/WTe_2_ FET biosensor with the immobilized probe DNA were chosen to demonstrate the specificity of the MoS_2_/WTe_2_ FET biosensors. Figure 4E,F show that there is no obvious current change after adding 1 fM N_1_–N_4_ samples, and the inset of Figure 4E is the current after local magnification. The complete current diagram after adding the N_1_–N_4_ samples is shown in Figure S6. In addition, there is a higher response after adding the 10 aM target DNA than that after adding 1 fM N_1_–N_4_ samples. Figure 4F shows a comparison of the response ratios between the target DNA and non-target DNA samples, with N_1_, N_2_, N_3_, and N_4_ representing non-target DNA samples and P representing the target DNA sample. Thus, the proposed MoS_2_/WTe_2_ FET biosensor has a good specificity. As shown in Figure 4G, the biosensing performance of the MoS_2_/WTe_2_ FET biosensor did not decrease significantly after sixty days and the peak to valley value (PV) was still within an acceptable error range (PV = 9.86%). Thus, the MoS_2_/WTe_2_ FET biosensor demonstrated excellent stability and durability.

### 3.5. Comparison of Similar Structures

**Figure 5:**
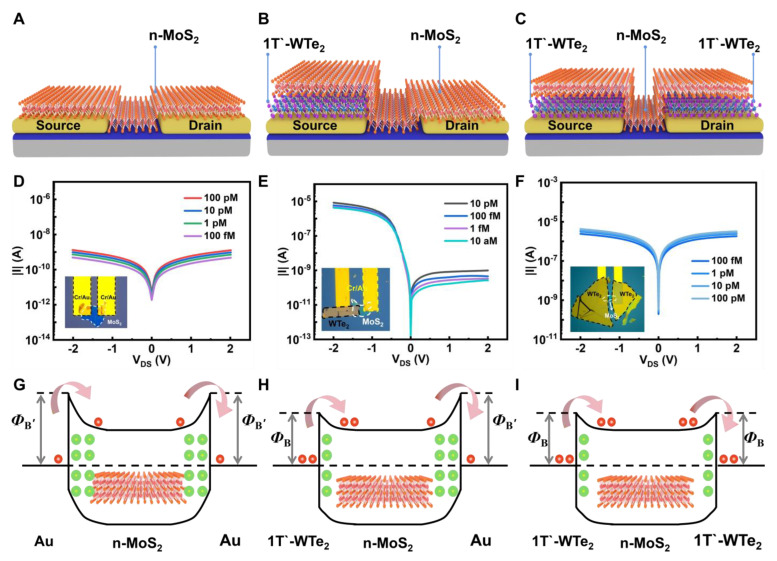
Comparison of similar structures. Schematic diagrams (**A**–**C**), output characteristic curves (**D**–**F**), and band diagrams (**G**–**I**) of the Au/MoS_2_/Au, WTe_2_/MoS_2_/Au, and WTe_2_/MoS_2_/WTe_2_ structures, respectively.

The advantage of the “rectified signal” (rectification ratio) was demonstrated by constructing three kinds of FETs: Au/MoS_2_/Au FET (Figure 5A), WTe_2_/MoS_2_/Au FET (Figure 5B), and WTe_2_/MoS_2_/WTe_2_ FET (Figure 5C). Except for the reported WTe_2_/MoS_2_/Au FET, the other two FETs only showed “absolute value signal” (source-drain current) as the response signal. Due to the presence of a large symmetric barrier in Au/MoS_2_/Au FET [44], the obtained current was about 1 × 10^−10^ A, as shown in Figure 5D. In contrast, due to the presence of an asymmetric barrier in WTe_2_/MoS_2_/Au FET, the forward source-drain current dominated by MoS_2_/Au junction was about 1 × 10^−10^ A, while the reverse source-drain current dominated by WTe_2_/MoS_2_ junction was about 1 × 10^−6^ A (See Figure 5E). Furthermore, Figure 5F shows that, due to the presence of a symmetric small-barrier WTe_2_/MoS_2_/WTe_2_ FET, the source-drain current was about 1 × 10^−6^ A. Figure S7 shows the changes in the transfer characteristic curves after introducing biomolecules in three different structures.

The source-drain current signals of the three structures were very weak, but the rectification ratio signal of the WTe_2_/MoS_2_/Au FET biosensor had significant variations. This indicates that the rectification ratio signal effectively amplifies the molecular signal variation and improves molecular signal sensitivity. Figure 5G–I shows the band diagram of the three structures. When the target biomolecules modified the sensing materials, the Schottky barrier widths were modulated. As shown in Figure 5G, when the target DNA caused electron doping [22], the Schottky barrier width of the MoS_2_/Au junction decreased and the source-drain current increased. As shown in Figure 5H, the asymmetric barrier decreased after adding the target DNA, resulting in an increased current and a decreased rectification ratio (Figure S8). Figure 5I shows that, when the target DNA was added into WTe_2_/MoS_2_/WTe_2_ FET, it caused electron doping and decreased the Schottky barrier width, increasing the source-drain current.

Moreover, the rectification ratio signal of the WTe_2_/MoS_2_/Au FET biosensor is stable for long periods of time (Figure S9), indicating that the “rectified signal” (the rectification ratio) is not affected by the solution environment and is a promising biosensing signal in FET biosensors. 

## 4. Conclusions

This study developed a novel strategy using the “rectified signal” in the output characteristic curve to detect biomolecules. The strategy effectively avoided the interference of external factors and was suitable for the quantitative analysis of trace targets in complex biological systems. Based on this strategy, a highly reliable and stable MoS_2_/WTe_2_ FET biosensor with an asymmetric Schottky barrier was developed. The experiments showed that the prepared biosensor achieved an ultra-sensitive and specific detection of the DYRK1A gene in the range of 10 aM–100 pM, indicating that the MoS_2_/WTe_2_ FET biosensor has great application potential in the prenatal screening of Down syndrome. This study demonstrated the importance of using a “rectified signal” in FET biosensors and provided a novel perspective for designing and optimizing biosensors based on two-dimensional materials.

## Data Availability

Data are contained within the article and Supplementary Materials.

## Supplementary Materials

The following supporting information can be downloaded at: https://www.mdpi.com/article/10.3390/nano14020226/s1. Figure S1: Fabrication process diagram of the MoS_2_/WTe_2_ FET biosensor; Figure S2: The functionalization and immobilization process of the device; Figure S3: High-resolution XPS spectra of Mo 3d and S 2p; Figure S4: EDS images mentioned in Figure 3B; Figure S5: The current results of different concentrations of the target DNA (DYRK1A) under (A) positive and (B) negative bias voltages; Figure S6: The complete current diagram after adding the N_1_–N_4_ samples; Figure S7: The transfer characteristic curves of the Au/MoS_2_/Au, WTe_2_/MoS_2_/Au, and WTe_2_/MoS_2_/WTe_2_ structures; Figure S8: Output characteristic curves measured at different biomolecular concentrations in the MoS_2_/WTe_2_ FET biosensor; Figure S9: Stability comparison of the “rectified signal” and “absolute value signal”; Table S1: The DNA sequences purchased from Sangon Biotech (Shanghai) Co., Ltd.; Table S2: Comparison of the biosensing performances of the various FET biosensors. References [45,46,47,48,49,50,51,52] are cited in the supplementary materials.
