# Supplementary material for: Asymmetric Schottky Barrier-Generated MoS2/WTe2 FET Biosensor Based on a Rectified Signal
Source: Nanomaterials (Basel). 2024 Jan 20;14(2):226. doi: 10.3390/nano14020226 (PMC10820193; doi:10.3390/nano14020226)
Supplement: Supplementary file 1 [file nanomaterials-14-00226-s001.zip › nanomaterials-2810290-supplementary.pdf]

Supporting Materials

Asymmetric Schottky Barrier-Generated MoS_2/WTe_2 FET Biosensor Based on a Rectified Signal

Xinhao Zhang [1,†], Shuo Chen [1,†], Heqi Ma [1], Tianyu Sun [1], Xiangyong Cui [1], Panpan Huo [1], Baoyuan Man [1,*] and Cheng Yang [1,2,*]

[1] School of Physics and Electronics, Shandong Normal University, Jinan 250014, China; zhangxh0050@163.com (X.Z.); 1091471811@qq.com (S.C.); 1005740998@qq.com (H.M.); 377633468@qq.com (T.S.); 2199491415@qq.com (X.C.); ghost54051@qq.com (P.H.)

[2] Shandong Provincial Engineering and Technical Center of Light Manipulations, Shandong Normal University, Jinan 250014, China

* Correspondence: byman@sdnu.edu.cn (B.M.); chengyang@sdnu.edu.cn (C.Y.)

† These authors contributed equally to this work.

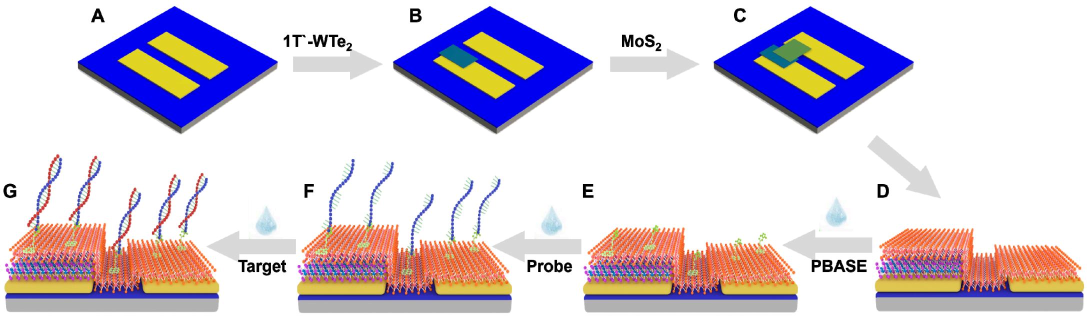

Figure S1. Fabrication process diagram of MoS_2/WTe_2 FET biosensor. (A) A clean and tidy Si/SiO_2 substrate is prepared. (B) WTe_2 is transferred by using a two-dimensional material transfer platform. (C) MoS_2 is transferred by using a two-dimensional material transfer platform. (D) A MoS_2/WTe_2 Schottky heterojunction is formed. (E) Add PBASE on the MoS_2/WTe_2 FET biosensor. (F) Add probe DNA on the MoS_2/WTe_2 FET biosensor. (G) Add target DNA on the MoS_2/WTe_2 FET biosensor.

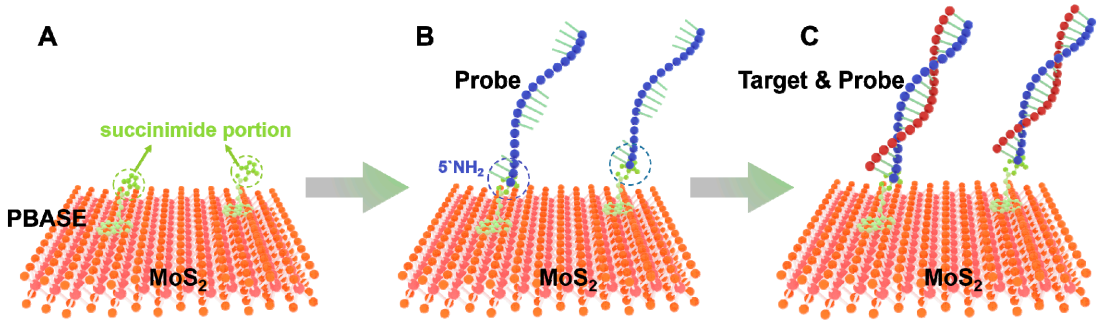

Figure S2. The functionalization and immobilization process of the device. (A) PBASE, when dissolved in DMSO, stacks its pyrene group on the surface of MoS_2, binds to MoS2. (B) The succinimidyl ester group of PBASE is connected to the amino-modified probe DNA through a cross-linking reaction. (C) The target DNA is combined with the probe DNA through complementary base pairing.

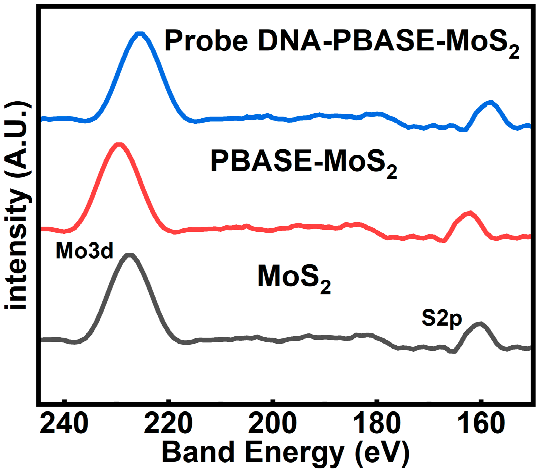

Figure S3. High-resolution XPS spectra of Mo 3d, S 2p. The movement of Mo 3d peak and S 2p peak confirmed that PBASE and probe DNA were successfully combined on the surface of MoS_2/WTe_2 FET biosensor.

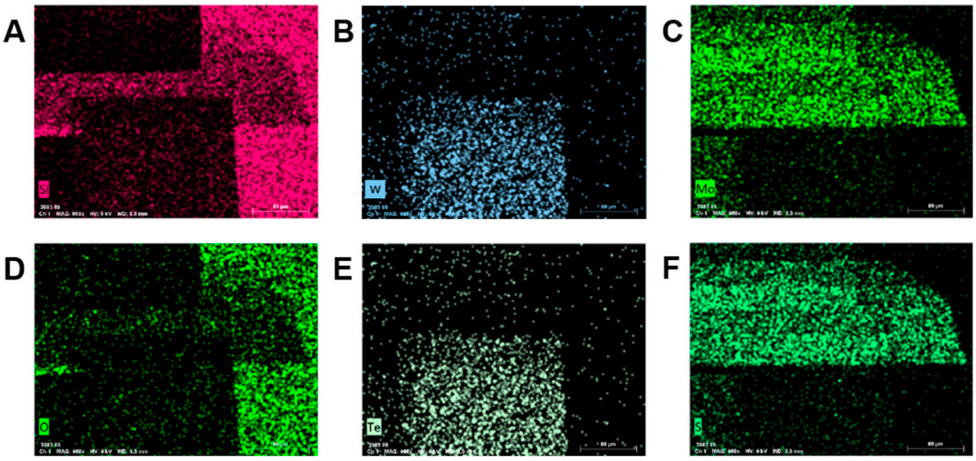

Figure S4. EDS images mentioned in Figure 3B. The above EDS images represent (A) Si, (B) W, (C) Mo, (D) O, (E) Te and (F) S elements respectively.

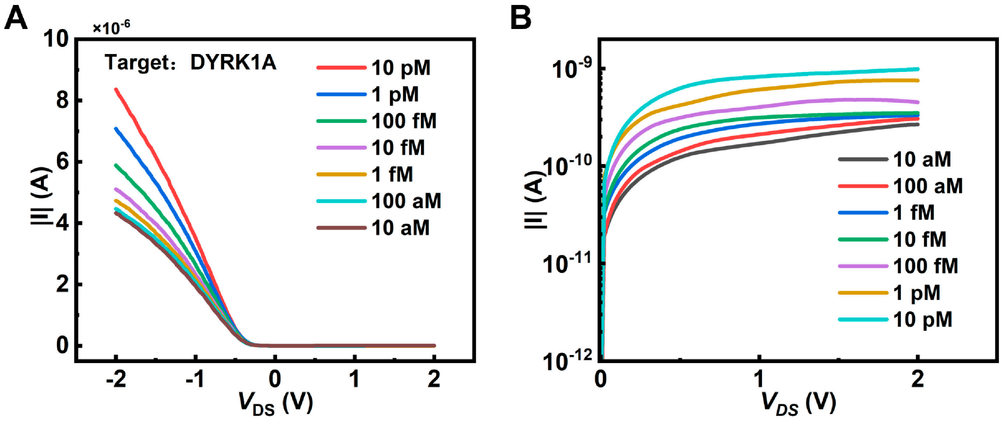

Figure S5. The current results of different concentrations of target DNA (DYRK1A) under (A) positive and (B) negative bias voltages.

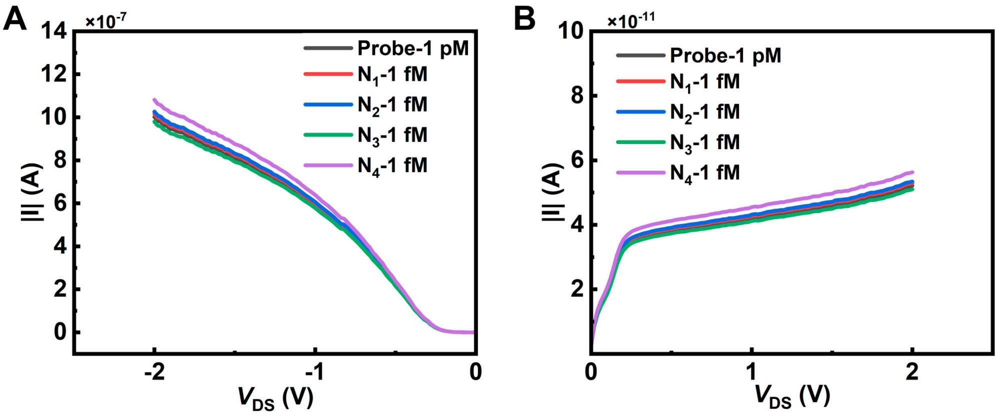

Figure S6. The complete current diagram after adding N_1-N_4 samples. Source-drain current under (A) negative bias and (B) positive bias in linear scale.

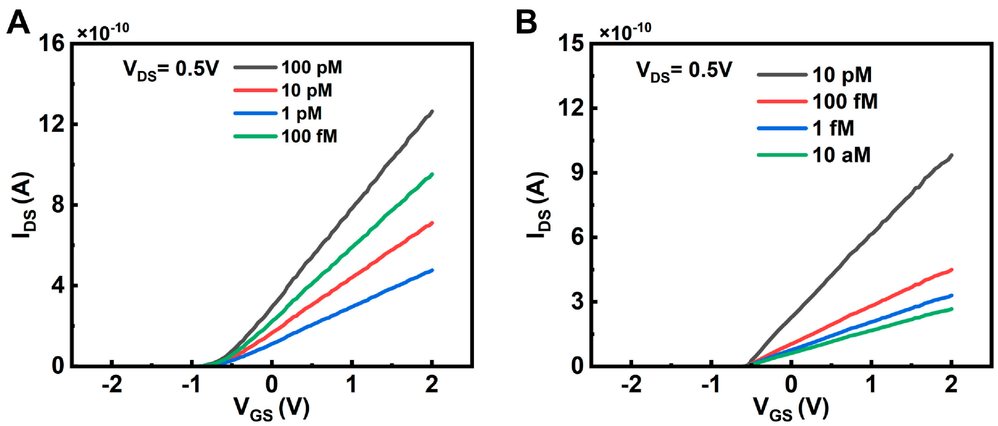

Figure S7: The transfer characteristics curves of the (A) Au/MoS_2/Au and (B) WTe_2/MoS_2/Au structures, respectively.

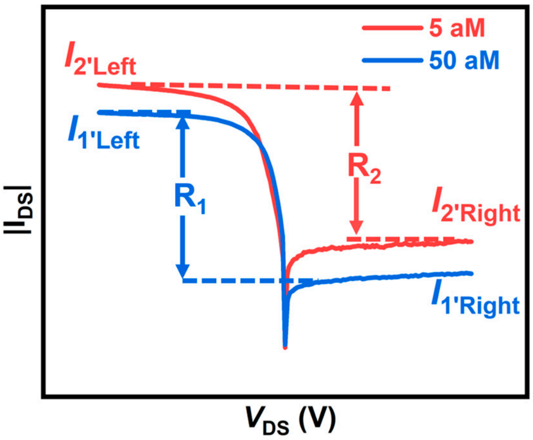

Figure S8. Output characteristics curves measured at different biomolecular concentrations in the MoS_2/WTe_2 FET biosensor. I_1 and I_2 respectively represent the source-drain current at different concentrations, I_{Left} and I_{Right} respectively represent the source-drain current dominated by different Schottky barriers, R_1 and R_2 respectively represent the rectification ratio of MoS_2/WTe_2 FET biosensor at different concentrations

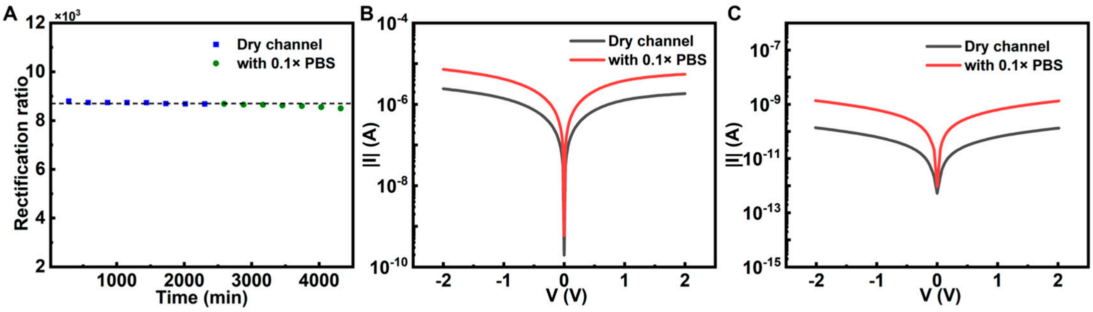

Figure S9. Stability comparison of "rectified signal" and "absolute value signal". The output current curves of (A) WTe_2/MoS_2/Au FET, (B) $WTe_2/MoS_2/WTe_2$ FET, and (C) Au/MoS_2/Au FET under external factors such as water molecules, respectively. Under external factors such as water molecules, the rectification ratio signal of the WTe_2/MoS_2/Au FET biosensor is stable within long times, as shown in Supplementary Figure 3A.

Table S1. The DNA sequences purchased from Sangon Biotech (Shanghai) Co., Ltd

Names	Sequences (5′→3’)
Probe DNA in Figs. 1B-1C, 1H-1I, 2I, 3B-3F	NH_2-C_6-CAGAGTCACTGACTCACTA
Target DNA in Figs. 1C,1I,2I,2B-2D	TTAGTGAGTCAGTGACTCTG
RNA in Figs. 3F-3G	CAGGUGGAACCUCAUCAGGAGAUGC
T1 in Figs. 3F-3G	TTTTTTTTTTUUUUUUCCCGTCCGTATGG
T2 in Figs. 3F-3G	TCTCAAGGACCACCGCATCTCTACCCATACGGACGGG
3-base Mismatch DNA in Figs. 3F-3G	TTAGTGAGTCAGTGACTGAC

Target DNA sequence in Figs. 1C,1I,2I,2B-2D was selected from GeneBank: NG_009366.2

Table S2. Comparison of biosensing performance among various FET biosensors

Sensing materials	Functionalization methods	Probe types	Target biomarkers	LOD	Signal types	Ref
Graphene	PBASE	ssDNA	Antigen	2.6 pM	V_{Dirac}; I_{DS}	[1]
rGO	PBASE	Antibody	Antigen	0.1 pg/mL	V_{Dirac}	[2]
Graphene	AuNPs	ssDNA	20-mer DNA	15 aM	V_{CNP}	[3]
Mxenes/graphene	APTES	Antibody	Antigen	1 fg/mL	V_G; I_{DS}	[4]
MoS_2	Physically adsorb	Antibody	Antigen	1 pM	V_{th}	[5]
MoS_2	AuNPs	ssDNA	30-mer DNA	10 aM	I_{DS}	[6]
rGO	Physically adsorb	ssDNA	48-mer DNA	5 pM	I_{DS}	[7]

Exfoliated-Graphene	EDC+NHS	Antibody	Antigen	10 fg/mL	Resist-ance	[8]
MoS_2/WTe_2	PBASE	ssDNA	20-mer DNA	10 aM	Recti-fication ratio	This work

References

1. Khan, N.I.; Mousazadehkasin, M.; Ghosh, S.; Tsavalas, J.G.; Song, E. An integrated microfluidic platform for selective and real-time detection of thrombin biomarkers using a graphene FET. *Analyst* **2020**, *145*, 4494-4503.
2. Park, D.; Kim, J.H.; Kim, H.J.; Lee, D.; Lee, D.S.; Yoon, D.S.; Hwang, K.S. Multiplexed femtomolar detection of Alzheimer's disease biomarkers in biofluids using a reduced graphene oxide field-effect transistor. *Biosens. Bioelectron.* **2020**, *167*, 112505.
3. Danielson, E.; Sontakke, V.A.; Porkovich, A.J.; Wang, Z.; Kumar, P.; Ziadi, Z.; Yokobayashi, Y.; Sowwan, M. Graphene based field-effect transistor biosensors functionalized using gas-phase synthesized gold nanoparticles. *Sens. Actuators. B: Chem* **2020**, *320*, 128432.
4. Li, Y.; Peng, Z.; Holl, N.J.; Hassan, M.R.; Pappas, J.M.; Wei, C.; Izadi, O.H.; Wang, Y.; Dong, X.; Wang, C. MXene–graphene field-effect transistor sensing of influenza virus and SARS-CoV-2. *ACS omega* **2021**, *6*, 6643-6653.
5. Park, H.; Lee, H.; Jeong, S.H.; Lee, E.; Lee, W.; Liu, N.; Yoon, D.S.; Kim, S.; Lee, S.W. MoS2 field-effect transistor-amyloid-β1–42 hybrid device for signal amplified detection of MMP-9. *Anal. Chem.* **2019**, *91*, 8252-8258.
6. Liu, J.; Chen, X.; Wang, Q.; Xiao, M.; Zhong, D.; Sun, W.; Zhang, G.; Zhang, Z. Ultrasensitive monolayer MoS2 field-effect transistor based DNA sensors for screening of down syndrome. *Nano Lett.* **2019**, *19*, 1437-1444.
7. Chan, C.; Shi, J.; Fan, Y.; Yang, M. A microfluidic flow-through chip integrated with reduced graphene oxide transistor for influenza virus gene detection. *Sens. Actuators. B: Chem* **2017**, *251*, 927-933.
8. Islam, S.; Shukla, S.; Bajpai, V.K.; Han, Y.-K.; Huh, Y.S.; Kumar, A.; Ghosh, A.; Gandhi, S. A smart nanosensor for the detection of human immunodeficiency virus and associated cardiovascular and arthritis diseases using functionalized graphene-based transistors. *Biosens. Bioelectron.* **2019**, *126*, 792-799.
